# Structural characterization of chaos game fractals using small-angle scattering analysis

**DOI:** 10.1371/journal.pone.0181385

**Published:** 2017-07-13

**Authors:** Eugen Mircea Anitas, Azat Slyamov

**Affiliations:** 1 Joint Institute for Nuclear Research, Dubna, Moscow region, Russian Federation; 2 Horia Hulubei National Institute of Physics and Nuclear Engineering, Bucharest-Magurele, Romania; 3 Institute of Nuclear Physics, Almaty, Kazakhstan; Duke University, UNITED STATES

## Abstract

Small-angle scattering (SAS) technique is applied to study the nano and microstructural properties of spatial patterns generated from chaos game representation (CGR). Using a simplified version of Debye formula, we calculate and analyze in momentum space, the monodisperse scattering structure factor from a system of randomly oriented and non-interacting 2D Sierpinski gaskets (SG). We show that within CGR approach, the main geometrical and fractal properties, such as the overall size, scaling factor, minimal distance between scattering units, fractal dimension and the number of units composing the SG, can be recovered. We confirm the numerical results, by developing a theoretical model which describes analytically the structure factor of SG. We apply our findings to scattering from single scale mass fractals, and respectively to a multiscale fractal representing DNA sequences, and for which an analytic description of the structure factor is not known *a priori*.

## Introduction

A multitude of artificial and natural processes ranging from nano to macro scales generate self-similar [1] structures, which means that they look exactly or approximately similar to a part of themselves under a transformation of scale [2–7]. These objects are referred to as deterministic fractals (Cantor sets, Koch snowflake, Sierpinski gasket; SG) if the structure is exactly self-similar, or random fractals (polymers, percolation clusters, surfaces, DNA sequences) if the structure is statistically self-similar [8, 9]. At nano and micro scales, the self-similarity plays an important role in the electromagnetic [10], optical [11, 12] or dynamical [13] properties, and thus one of the main tasks is to understand the correlation between fractal microstructure and its physical properties [14].

The main structural property of fractals with a single scaling factor is the fractal (Hausdorff) dimension, which can be rigorously defined mathematically [15]. For mass fractals [16], we adopt here a more simple definition of the dimension *D*_m_: $M\left(r\right)\propto r^{D_{m}}$, where *M*(*r*) is the mass inside a spherical surface of radius *r* [17]. For surface fractals [18], the fractal dimension D_*s*_ describes the area measured by covering the surface with the smallest possible number of balls with radius *r*, through the relation $S\left(r\right)\propto r^{2-D_{s}}$. For a smooth surface, the value D_*s*_ is obtained. Using the ball-covering process, we can see that the mass and the surface of a mass fractal are equivalent, and thus D_*m*_ = D_*s*_, while for a surface fractal D_*m*_ = *d* and *d* − 1 < D_*s*_ < *d*.

A very efficient experimental technique which can distinguish between mass and surface fractals is small-angle scattering (SAS; X-rays, neutrons, light) [19, 20], which yields the differential elastic cross-section per unit solid angle as a function of the momentum transfer, and describes the spatial density-density correlations in the investigated sample. The difference between mass and surface fractals is revealed through the value of the scattering exponent in the simple power-law decay of the scattering intensity *I*(*q*) ∝ *q*^−*τ*^, on a double logarithmic scale [16, 18, 21–24]. Experimentally, if *τ* < *d*, the sample is a mass fractal with dimension *τ* = D_m_, while if *d* < *τ* < *d* + 1, the sample is a surface fractal. The above conclusions hold also for generalized power-law decays given as a superposition of maxima and minima on a simple power-law decay, and which are specific to scattering from monodisperse deterministic fractals [24–29].

Theoretically, various fractal-generating algorithms are developed to reconstruct a large number of real self-similar patterns, from well-known deterministic fractals to various classes of disordered systems. A frequently used algorithm which allows a visual representation of both local and global patterns, is chaos game representation (CGR) [30], with important applications in investigating biological samples, such as gene structures [31, 32]. Technically, CGR is an iterative map that processes sequences of units in order to find their positions in a continuous space [32]. For structural investigations using SAS, CGR is very convenient because it gives directly the coordinates of the scattering centers, which can be used in an efficient expansion of the Debye formula, for calculating the SAS intensity spectrum [33].

Here, we show that for fractal models based on CGR and which have a single scaling factor, SAS allows us to reveal, besides standard fractal dimension, other structural properties such as the fractal scaling factor, the number of scattering units inside the fractal, fractal overall size and the minimal distance between the scattering units. For this purpose, we calculate both analytically and numerically the monodisperse scattering structure factor from a two-dimensional SG generated deterministically, and respectively by CGR. However, for a fractal with multiple scaling factors, the randomness leads to an almost complete smearing of the minima on the scattering curve, and thus, the scaling factors can hardly be recovered. This is in agreement with SAS curves obtained from mass random fractals built from deterministic ones [34].

## 1 Theoretical background

### 1.1 Small-angle scattering

Let’s consider a two-phase approximation where the microscopic scattering objects have scattering length *b*_*j*_ and scattering length density (SLD) *ρ*_s_(***r***) = ∑_*j*_ *b*_*j*_ *δ*(***r*** − ***c***_*j*_). Here, ***c***_*j*_ are the position vectors of the scattering objects, and *δ* is Dirac’s delta function. In a very good approximation we can neglect multiple scattering since in the case of fractal aggregates, due to such processes, the value of the fractal dimension is changed [35] and the scaling factor is lost [36]. Thus, the corresponding differential elastic cross section is defined by dσ/dΩ = |*A*_*t*_(***q***)|^2^, where *A*_*t*_(***q***) = ∫_*V*′_ *ρ*_*s*_(***r***) exp(*i****q*** ⋅ ***r***)d***r*** is the total scattering amplitude and *V*′ is the volume irradiated by the incident beam. If we consider that the fractal objects are immersed in a solid matrix of SLD *ρ*_0_, then the scattering contrast is defined by Δ*ρ* = *ρ* − *ρ*_0_, and the total scattering intensity will be given by

$$\begin{array}{c} I\left(q\right)\equiv \frac{1}{V^{{}^{\prime }}}\frac{\text{d}\sigma }{\text{d}\Omega }=c\;{\left|\Delta \rho \right|}^{2}\;V^{2}\;\langle {\left|F\left(q\right)\right|}^{2}\rangle , \end{array}$$ (1)

where *c* is the concentration of fractal, *V* is the volume of each fractal, and *F*(***q***) ≡ (1/*V*) ∫_*V*_ exp(−*i****q*** ⋅ ***r***)d***r*** is the normalized form factor, with *F*(0) = 1. The symbol 〈⋯〉 denotes the ensemble averaging over all orientations which, for an arbitrarily function *f*, is calculated according to $\langle f(q)\rangle =\left(1/4\pi \right)\int_{0}^{\pi }d\theta \sin\theta \int_{0}^{2\pi }d\phi f\left(q,\theta ,\phi \right)$. In what follows, since the fractals are two-dimensional, the volume *V* in Eq (1) shall be replaced by the corresponding surface area. Note that the above averaging procedure allows the rotation of the fractals in three-dimensional space, with equal probability.

Furthermore, if each fractal is composed of the same number *N* of identical scattering ‘units’, then its form factor is given by [25]

$$\begin{array}{c} F\left(q\right)=\rho_{q}F_{0}\left(qR\right)/N, \end{array}$$ (2)

where *F*_0_(*qR*) is the form factor of scattering units of size *R*, *ρ*_***q***_ = ∑_*j*_ exp(−*i****q*** ⋅ ***r***_*j*_) is the Fourier component of the density of units centers, and ***r***_*j*_ are the center-to-mass positions of units. Taking into account that the structure factor can be defined as

$$\begin{array}{c} S\left(q\right)\equiv \langle \rho_{q}\rho_{-q}\rangle /N, \end{array}$$ (3)

then the scattering intensity becomes [25]

$$\begin{array}{c} I\left(q\right)=I\left(0\right)S\left(q\right){\;|F_{0}\left(qR\right)|}^{2}/N. \end{array}$$ (4)

### 1.2 Iterated function systems

Iterated function systems (IFS) provide a useful framework for classification and description of fractals. By definition, a (hyperbolic) IFS consists of a complete metric space (**X**, *d*) together with a finite set of contraction mappings *w*_*n*_: **X** → **X**, with respective contractivity factors *s*_*n*_, *n* = 1, 2, ⋯, *N* [30]. Using a shorthand notation, an IFS is {**X**; *w*_*n*_, *n* = 1, 2, ⋯, *N*} and *s* = max{*s*_*n*_, *n* = 1, 2, ⋯, *N*}. We recall here that, in general, a transformation *f*: **X** → **X** on a metric space (**X**, *d*) is a contraction mapping if there is a constant (contractivity factor) 0 ≤ *s* < 1 such that

$$\begin{array}{c} d(f(x),f(y))\leq s\cdot d\left(x,y\right)\;\;\;\forall x,\;y\in X. \end{array}$$ (5)

The main property used here is that by considering a hyperbolic IFS with contractivity factor *s*, and (H(X),h(d)) denoting the space of nonempty compact subsets with the Hausdorff metric *h*(*d*), the transformation W:H(X)→H(X) defined by

$$\begin{array}{c} W\left(B\right)=\cup_{n=1}^{N}w_{n}\left(B\right),\;\forall B\in H\left(X\right), \end{array}$$ (6)

is a contraction mapping on the complete metric space (H(X),h(d)) with the contractivity factor *s* [30], i.e.

$$\begin{array}{c} h(W(B),W(C))\leq s\cdot h(B,C)\;\forall B,\;C\in H(X). \end{array}$$ (7)

Its unique fixed point, A∈H(X) obeys $A=\cup_{n=1}^{N}w_{n}\left(A\right)$, is given by *A* = lim_*m*→∞_ *W*^⚬*m*^(*B*) for any B∈H(X), and is called the *attractor* (or deterministic fractal) of the IFS [30].

For rendering pictures of attractors, and in order to calculate the corresponding structure factors from Eq (20), we will use a deterministic algorithm, based on the idea of computing a sequence of sets {*A*_*m*_ = *W*^⚬*m*^(*A*)} starting from an initial set *A*_0_, as well as a random iteration algorithm, based on ergodic theory. For simplicity, we restrict ourselves to IFS of the form $\left\{R^{2};\;w_{n},n=1,2,\cdots ,N\right\}$, where each mapping is an affine transformation.

Using the deterministic algorithm, we start by choosing a compact set $A_{0}\subset R^{2}$, and then we compute successively *A*_*m*_ according to the rule

$$\begin{array}{c} A_{m}=\cup_{n=1}^{N}w_{n}\left(A_{m-1}\right),\;for\;m=1,2,\cdots \end{array}$$ (8)

thus constructing the sequence $\{A_{m}:\;m=0,1,\cdots \}\subset H\left(X\right)$, which converges top the attractor of the IFS in the Hausdorff metric.

When using the random iteration algorithm, we start by assigning the probability *p*_*n*_ > 0 to *w*_*n*_ for *n* = 1, 2, ⋯, *N*, where $\sum_{n=1}^{N}p_{n}=1$. Then we choose a point *x*_0_ ∈ **X** and then we choose recursively, independently,

$$\begin{array}{c} x_{k}\in \{w_{1}\left(x_{k-1}\right),w_{2}\left(x_{k-1}\right)\cdots ,w_{N}\left(x_{k-1}\right)\}, \end{array}$$ (9)

where the probability of the event *x*_*k*_ = *w*_*n*_(*x*_*k* − 1_) is *p*_*n*_, and *k* = 1, 2, ⋯. This creates the sequence {*x*_*k*_: *k* = 0, 1, ⋯} ⊂ **X** which converges to the attractor of IFS.

## 2 Sierpinski gasket model

**Fig 1:**
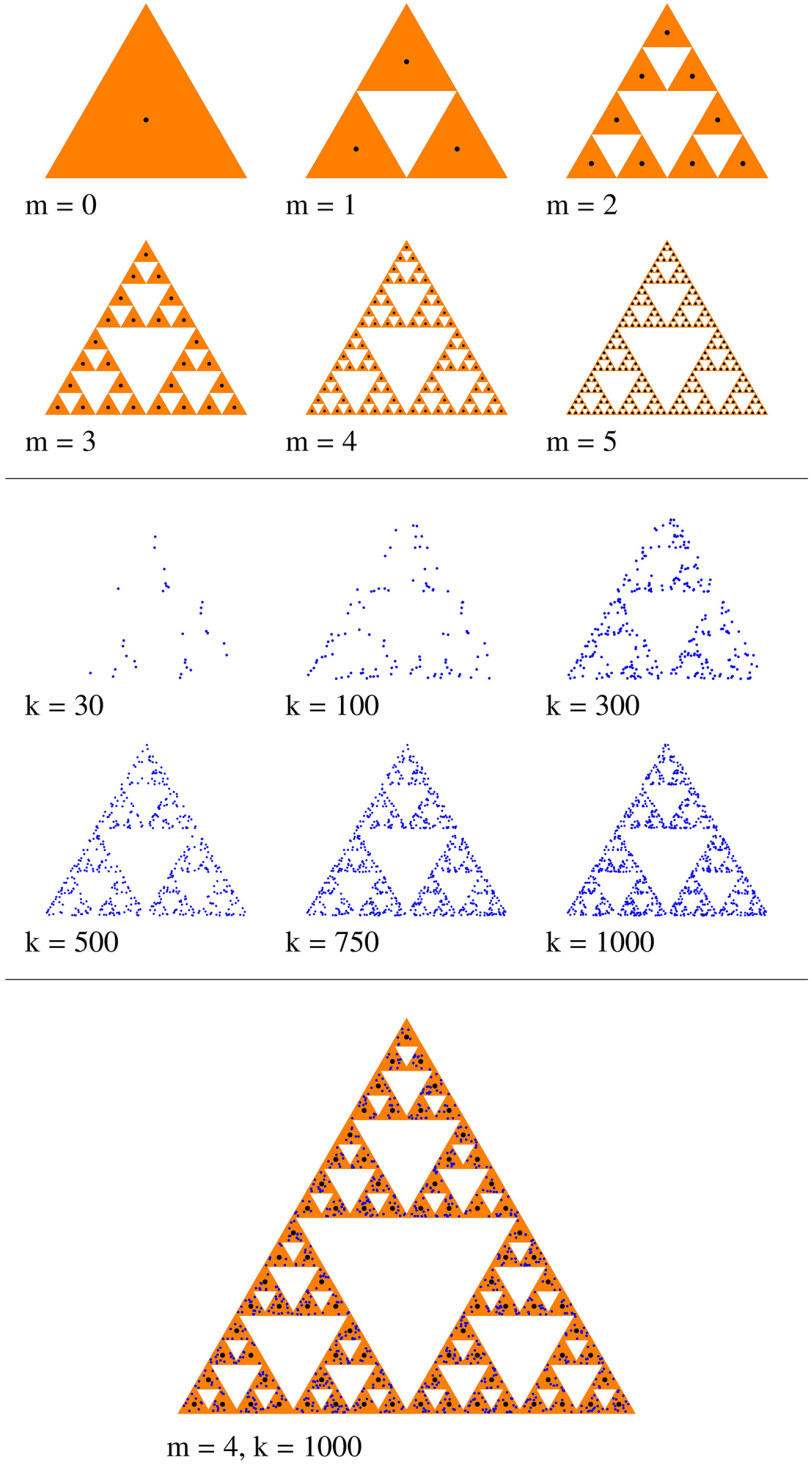
The structure of two-dimensional SG. Upper part: from deterministic algorithm up to *m* = 5 (black points); Middle part: from random iteration algorithm, for *k* = 30, 100, 300, 500, 750 and respectively *k* = 1000 points (blue); Lower part: a superposition of SG obtained from the two algorithms.

We consider a Sierpinski triangle of side length *a* = 1, and centered in the origin. Thus *N* = 3, and the matrix representation of the IFS of affine maps is

$$\begin{array}{c} w_{i}\;[\begin{array}{c} x\\ y \end{array}]=[\begin{array}{cc} a_{i} & b_{i}\\ c_{i} & d_{i} \end{array}][\begin{array}{c} x\\ y \end{array}]+[\begin{array}{c} e_{i}\\ f_{i} \end{array}] \end{array}$$ (10)

where the coefficients a_*i*_, b_*i*_, c_*i*_, d_*i*_, e_*i*_, f_*i*_ with *i* = 1, 2, 3 are given in Table 1. In the upper part of Fig 1 are shown the points (black) given by deterministic algorithm defined by Eq (10) up to iteration *m* = 5.

**Table 1:** The coefficients a_*i*_, b_*i*_, c_*i*_, d_*i*_, e_*i*_, f_*i*_ of the affine transforms *w*_*n*_ (first column) for deterministic algorithm in Eq (10), and the probabilities *p*_*n*_ (last column) for random iteration algorithm in Eq (9).

w	a	b	c	d	e	f	p
1	1/2	0	0	1/2	0	$1/(2\sqrt{3})$	1/3
2	1/2	0	0	1/2	-1/4	$-1/(4\sqrt{3})$	1/3
3	1/2	0	0	1/2	1/4	$-1/(4\sqrt{3})$	1/3

However, when using the random iteration algorithm, several ways of introducing randomness could be used. Here, we create the SG by playing chaos game [30] on three vertices which do not all lie on a line. The points of the SG fractal are obtained starting with an initial point chosen at random, and calculating each subsequent point as a fraction (here *β*_s_ = 1/2) of the distance between the previous point and one of the vertices (selected randomly at each iteration) of the triangle. By repeating this procedure for a large number of points, and selecting the vertex at random on each iteration, the SG is obtained. The peculiarity of the chaos game algorithm is that it plots points over the attractor in random order and with equal probabilities (see Table 1), as opposed to other methods which test each pixel to see whether it belongs to the fractal [30]. Fig 1, middle part, shows the result of chaos game for various values of the number of points *k*. The main feature shown is that by increasing *k*, leads to a better agreement with SG. One can notice that a value of *k* = 1000 is quite sufficient to generate a SG at *m* = 4 (see also Fig 1, lower part).

A comparison between SG obtained deterministically, and respectively using the random iteration algorithm is made also in Fig 1, lower part, which clearly shows that, excepting few dozen points which were omitted, all the others belong to the SG. In this way, a direct comparison between their respective structure factors can be performed (see next section).

## 3 Results and discussion

### 3.1 Structure factor of sierpinski gasket: Analytic representation

Let’s consider that the two-dimensional *SG* is constructed from equilateral triangles, as shown in Fig 1. At zero-*th* fractal iteration, the triangle is centered in the origin, it has the edge length *a*, and area $S^{T}=\sqrt{3}a^{2}/4$. At first iteration (*m* = 1), the initial triangle is divided into 4 smaller triangles, each of edge length *a*/2, the three triangles in the corners are kept, and the middle one is removed. At next iteration (*m* = 2) the same operation is repeated for each of the three triangles. At an arbitrary iteration *m*, one obtains the *SG* which consists of

$$\begin{array}{c} N_{m}=3^{m}, \end{array}$$ (11)

triangles of edge size *a*_*m*_ = *a*/2^*m*^. Thus, the fractal dimension is given by:

$$\begin{array}{c} D=\lim_{m\to \infty }\frac{\log N_{m}}{\log(a/a_{m})}\approx 1.585. \end{array}$$ (12)

The centers of these triangles coincide with the positions of the points in Fig 1 generated from IFS, using the deterministic algorithm (see previous section).

We can write the following product relation for the generative functions (*G*_*i*_(***q***)) of the *m*-th iteration of SG:

$$\begin{array}{c} P_{m}\left(q\right)\equiv \prod_{i=1}^{m}\;G_{i}\left(q\right), \end{array}$$ (13)

where the generative function at *m* = 1 is given by

$$\begin{array}{c} G_{1}\left(q\right)=\frac{1}{3}\;\sum_{k=0}^{2}\;e^{-iq\cdot b_{k}}, \end{array}$$ (14)

and $G_{m}\left(q\right)=G_{1}\left(\beta_{s}^{m-1}q\right)$. The translation vectors are given by

$$\begin{array}{c} b_{k}=\frac{a\sqrt{3}}{6}\{\cos(\frac{\pi }{3}\left(2k+\frac{3}{2}\right)),\sin(\frac{\pi }{3}\left(2k+\frac{3}{2}\right))\}, \end{array}$$ (15)

and the scaling factor is *β*_s_ = 1/2. Using Eq (2) without the term *F*_0_(*qR*), together with Eq (13) we can write that $\rho_{q}^{\left(m\right)}=N_{m}P_{m}\left(q\right)$, and from Eq (3), we obtain

$$\begin{array}{c} S_{m}\left(q\right)/N_{m}=\langle \prod_{i=1}^{m}\;{\left|G_{i}\left(q\right)\right|}^{2}\rangle \end{array}$$ (16)

where *G*_1_(***q***) is given by Eq (14). Thus, by using Eqs (4) and (16), the total intensity is

$$\begin{array}{c} I_{m}\left(q\right)/I_{m}\left(0\right)=S_{m}\left(q\right)/N_{m}, \end{array}$$ (17)

which is equivalent with Eq (4) but without the term *F*_0_(*qR*) since we are interested only in the contribution of the structure factor.

**Fig 2:**
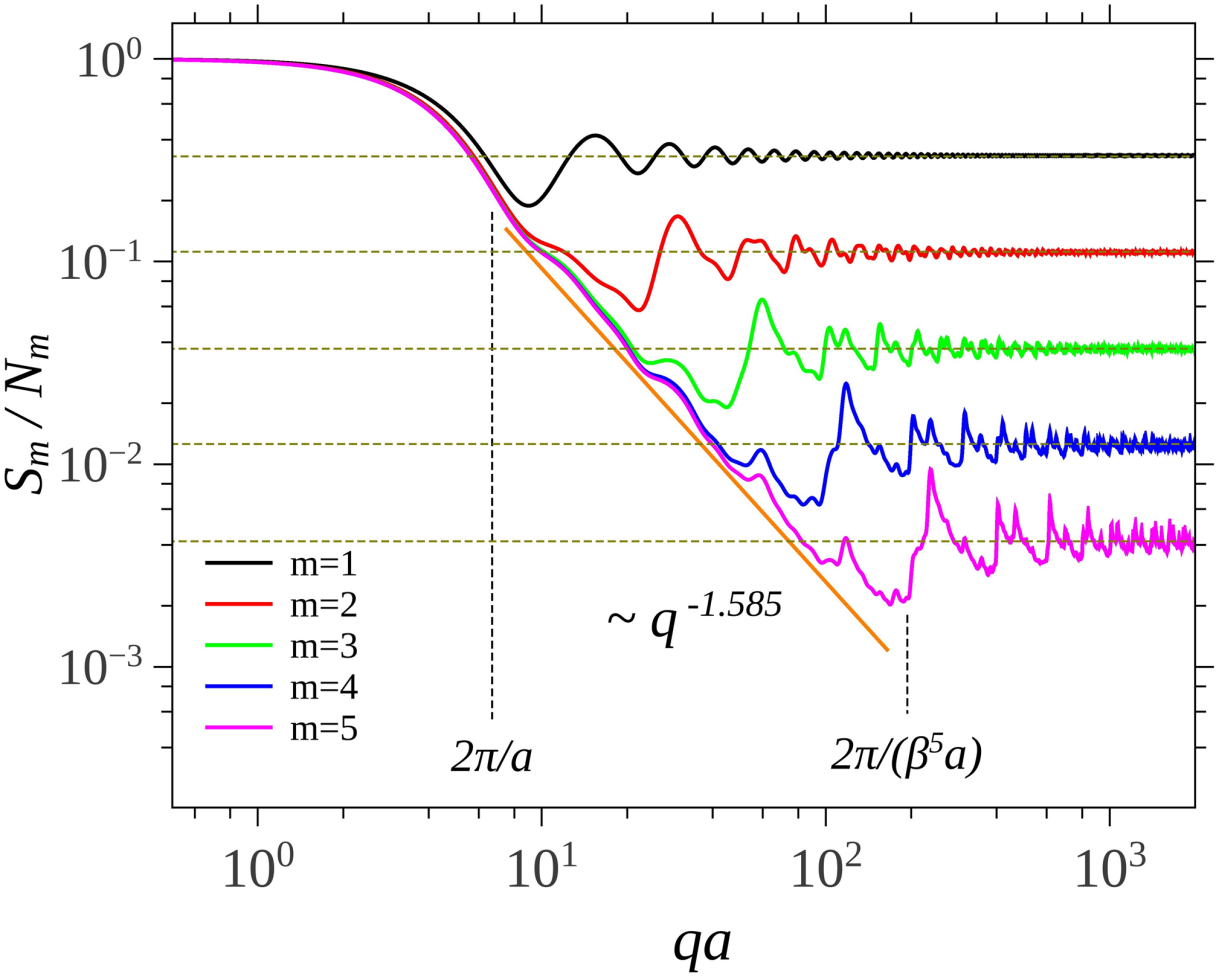
Structure factor for the first 5 iterations (*m* = 1, ⋯, 5) of the SG. Vertical lines indicate the limits of the fractal region at *m* = 5. Horizontal lines indicate the value of the asymptotes in the limit of high *q* values.

The scattering structure factors are shown in Fig 2. Generally, the main properties of the curves are kept as for the case of scattering from Vicsek, Cantor or Koch fractals [25, 27, 29]. Since the overall size of SG is about *a*, the Guinier range extends up to about 2*π*/*a*. This is followed by a fractal range up to $2\pi /(\beta_{s}^{m}a)$, and then the asymptotic region is attained. The fractal region is characterized by a superposition of maxima and minima on a power-law decay with the exponent *τ* = 1.585, which is in agreement with the theoretical value given by Eq (12). As expected, the values of the asymptotic values at high *q*, tend to 1/*N*_*m*_.

### 3.2 Structure factor of sierpinski gasket: Chaos game representation

**Fig 3:**
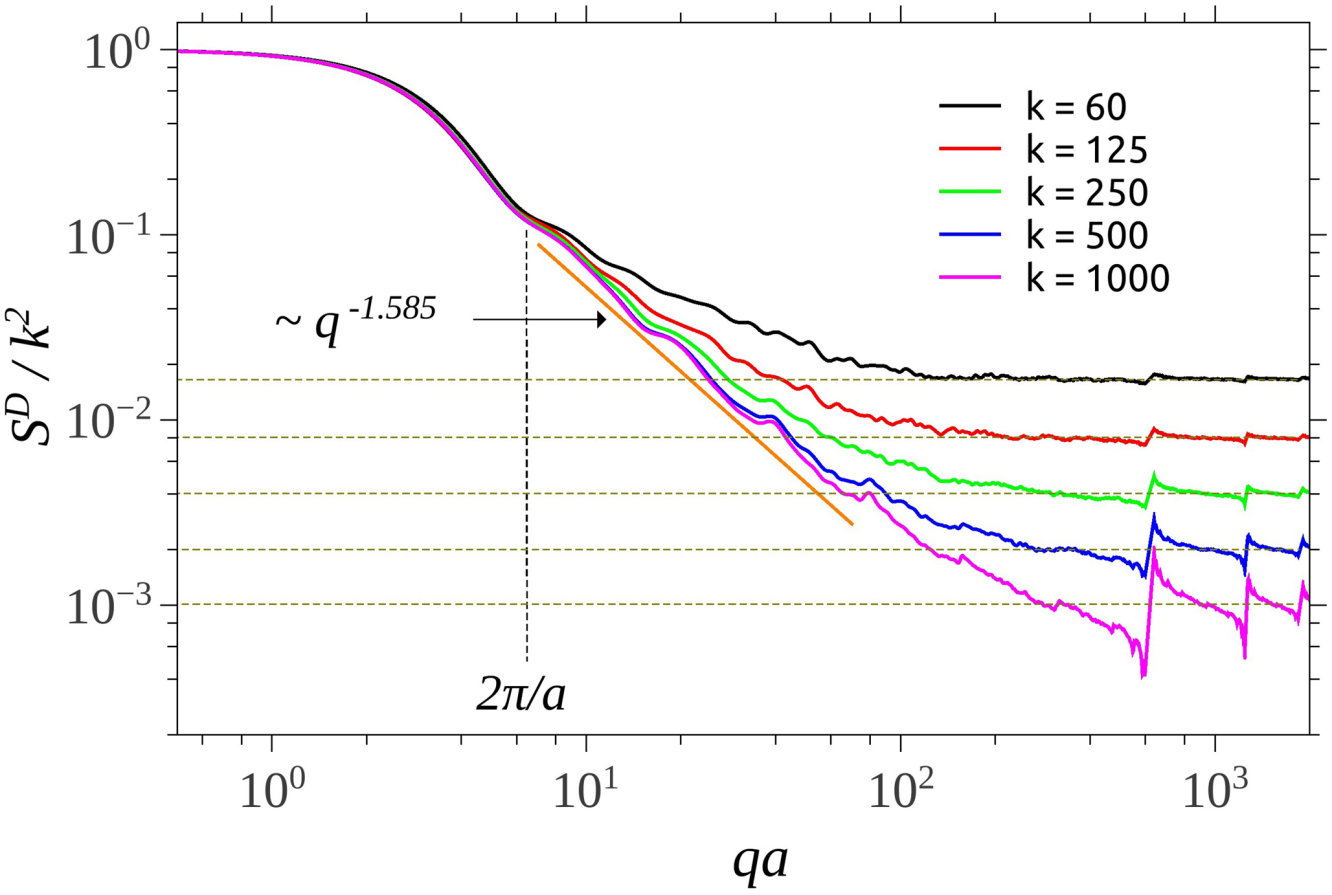
Structure factor for different values of number of units *k* of the SG using CGR. The vertical dotted line indicate the beginning of the fractal region. The horizontal lines indicate the value of the asymptotes in the limit of high *q* values.

Since we employ a CGR to generate random positions of *k* units inside each fractal, computationally, for monodisperse systems, we can start with the Debye formula [37]

$$\begin{array}{c} I^{D}\left(q\right)=kI_{s}\left(q\right)+2F_{s}{\left(q\right)}^{2}\;\sum_{i=1}^{k-1}\sum_{j=i+1}^{k}\;\frac{\sin qr_{ij}}{qr_{ij}}, \end{array}$$ (18)

where *I*_s_(*q*) is the intensity scattered by each fractal unit, and *r*_*ij*_ is the distance between units *i* and *j*. When the number of units exceeds few thousands, the computation of the term sin(*qr*_*ij*_)/(*qr*_*ij*_) is very time consuming, and thus it is handled via a pair-distance histogram *g*(*r*), such as in Fig 3 and below, with a bin-width commensurate with the experimental resolution [33]. Therefore Eq (18) can be rewritten as

$$\begin{array}{c} I^{D}\left(q\right)=kI_{s}\left(q\right)+2F_{s}^{2}\left(q\right)\sum_{i=1}^{N_{\text{bins}}}\;g\left(r_{i}\right)\frac{\sin qr_{i}}{qr_{i}}, \end{array}$$ (19)

where *g*(*r*_*i*_) is the pair-distance histogram at pair distance *r*_*i*_. The latter quantity is calculated from the positions of scattering units inside the fractal, according to the algorithm described in Sec. III. For determining fractal properties we can neglect the form factor, and consider $I_{s}\left(q\right)=F_{s}^{2}\left(q\right)=1$. Thus, the intensity given by Eq (19) becomes

$$\begin{array}{c} I^{D}\left(q\right)\equiv S^{D}\left(q\right)=k+2\;\sum_{i=1}^{N_{\text{bins}}}\;g\left(r_{i}\right)\frac{\sin qr_{i}}{qr_{i}}, \end{array}$$ (20)

and gives the structure factor of the fractal. Taking into account the normalization used in Eq (4), subsequently, the final expression of the scattering structure factor in Eq (20), is represented as *S*^*D*^(*q*)/*k*^2^.

**Fig 4:**
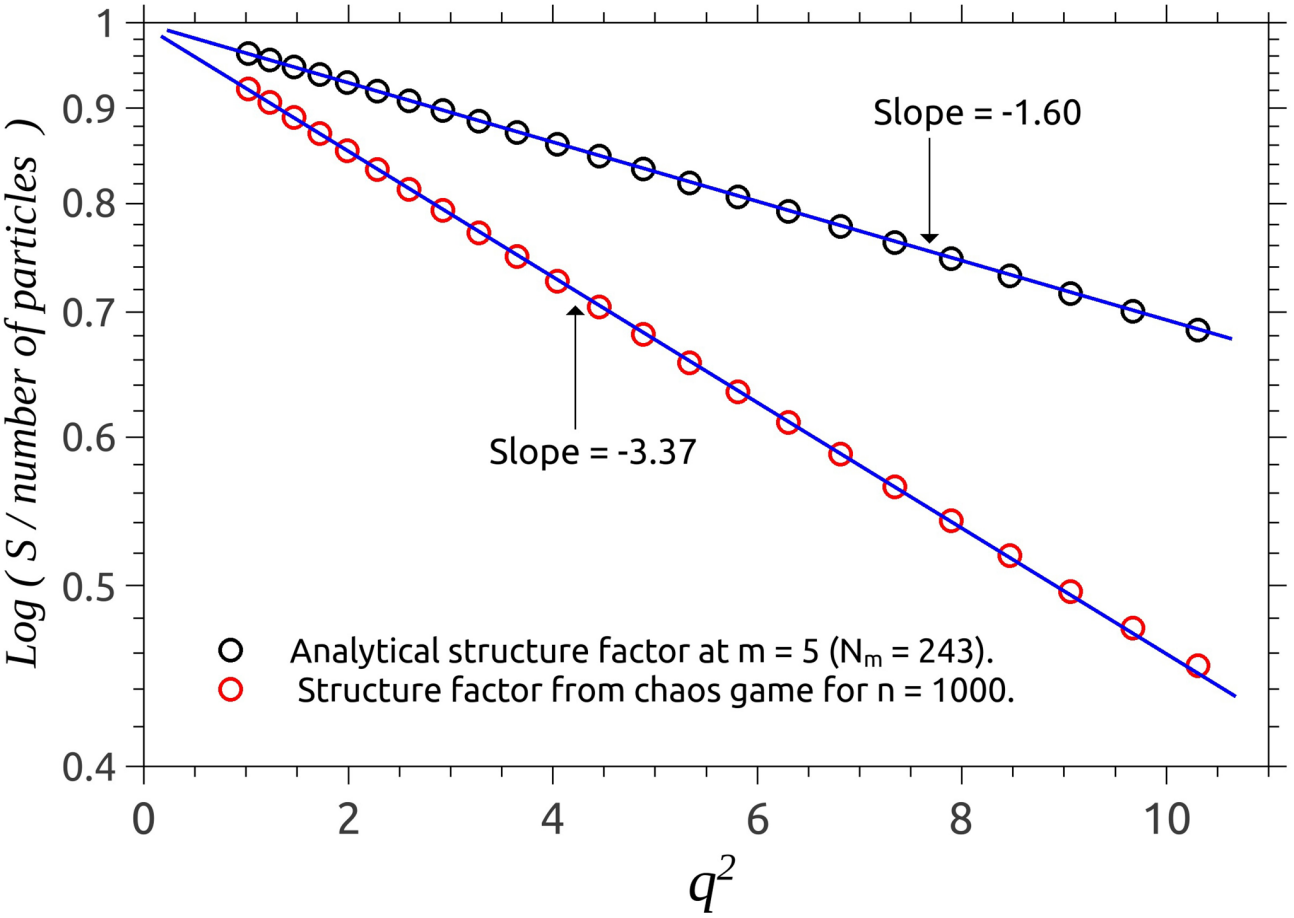
Guinier plot for the structure factors. Analytic representation (black), and CGR (red).

Fig 3 shows the scattering structure factor of SG built from the CGR, for different values of the number of units composing the fractal. All the curves show the three main regions characteristic to SG, obtained using the analytic representation (Fig 2). However, in the case of CGR, a transition region appears between fractal and asymptotic regions. For *k* = 1000 this region is at 10^2^ ≤ *qa* ≤ 8 ⋅ 10^2^ (Fig 3), and it arises due to the fact that in this range the pair distribution function does not approximate anymore a period-like pattern with a power-law distribution of the number of distances, as in the case of deterministic mass fractals [25]. Although the corresponding radius of gyration of SG from CGR is different as compared with the analytic representation (see Fig 4), the main features are that the length of fractal region increases with the number of scattering units, and the corresponding scattering exponents give the proper values of the fractal dimension. Note that, since *S*^*D*^ → *k* at high values of *q*, then *S*^*D*^/*k*^2^ → 1/*k*, which is in very good agreement with theoretical predictions.

### 3.3 Radii of gyration for analytic and chaos game representations

A direct comparison between the structure factors of SG using analytic and chaos game representations, requires a particular analysis, due to the algorithms involved in their construction.

As lower part of Fig 1 shows, the radius of gyration of SG from CGR ($R_{g}^{\text{CGR}}$) is slightly bigger than the radius of gyration obtained analytically ($R_{g}^{\text{AN}}$), due to the fact that in former case, some of the scattering units (blue) are situated further away from the center of the fractal, than the scattering units of SG from the analytic representation (black). In order to find the exact amount by which the overall size of these two representations differ, we use a Guinier plot which involves plotting log *S*(*q*) vs. *q*^2^ in order to obtain the slope $R_{g}^{2}/3$ (Fig 4). Numerical values for the slopes gives $R_{g}^{\text{CGR}}≃3.18$ and $R_{g}^{\text{AN}}≃2.19$, and thus $R_{g}^{\text{CGR}}/R_{g}^{\text{AN}}≃1.45$. These values have been calculated for a relatively high number of iterations (*m* = 5), and respectively, of number of particles (*k* = 1000), which assures a very good approximation for the ratio $R_{g}^{\text{CGR}}/R_{g}^{\text{AN}}$.

Now, by shifting the analytic curves to the right by the factor 1.45 determined above, we obtain an exact superposition of scattering curves in the Guinier region, for both analytic and chaos game representations, and a further analysis can be performed (see next section).

### 3.4 Structure factors of sierpinski gasket: Analytic versus chaos game representations

**Fig 5:**
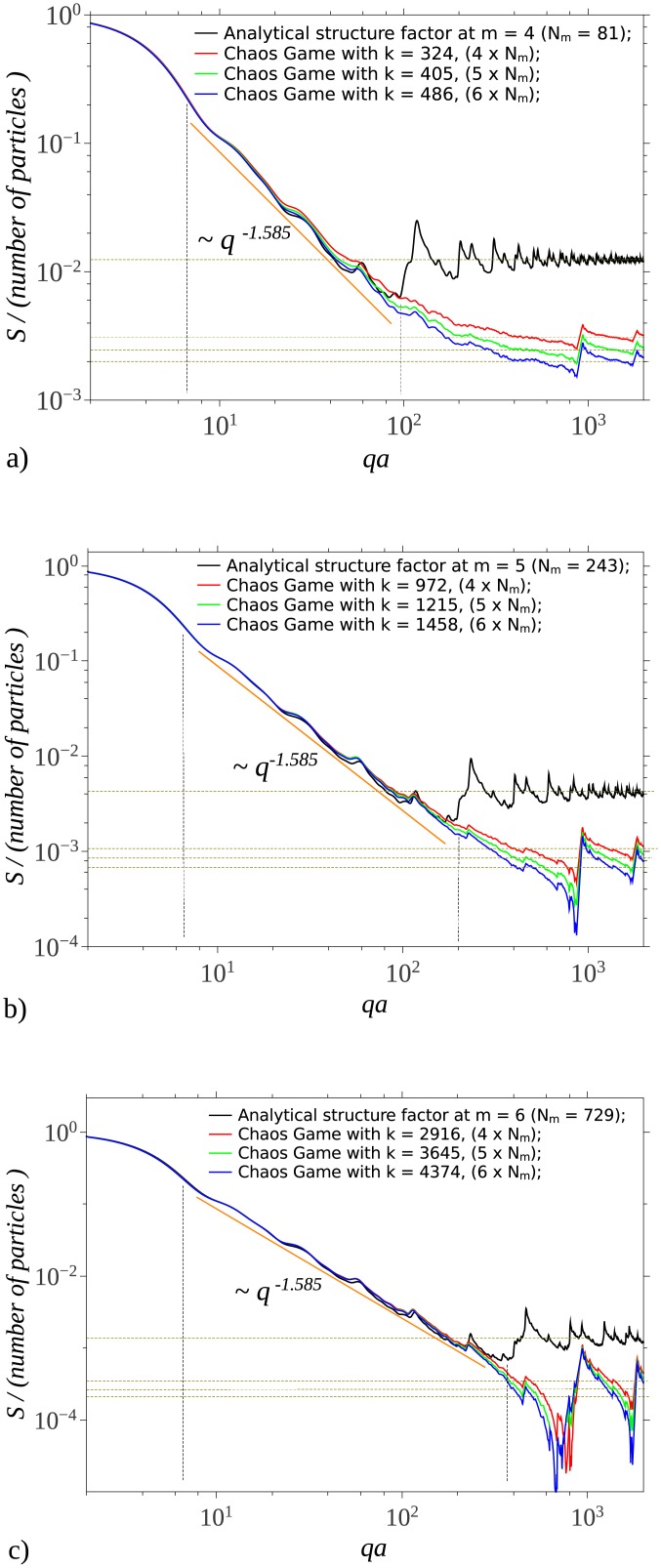
Structure factor of SG. Analytic and chaos game representations, at different iterations *m*, and respectively for various number of units. (a) *m* = 4; (b) *m* = 5; (c) *m* = 6. Vertical lines denote the beginning, and respectively the end of the fractal region corresponding to the analytic representation. Horizontal lines denote the asymptotic values at high *q*.

The scattering structure factors of SG are shown in Fig 5, for iteration number *m* = 4, 5, 6 (and thus for *N*_*m*_ = 81, 243, 729), and for different values of the number of scattering units *k*. For a given value of iteration number *m*, the left border of the fractal region is determined by the overall size of the fractal, while the right border is determined by the smallest distances between scattering units inside the fractal. This region is delimited by vertical lines in Fig 5. An important property is that under proper conditions the scattering curves obtained using CGR, can reproduce the analytic fractal region, and this shows that the fractal dimensions of chaos game fractals can also be obtained with good accuracy from SAS data. We found numerically that for 3*D* SG, the fractal region and fractal dimension of the analytic structure factor are reproduced when the minimum number of particles is about *k* ≃ 4 ⋅ *N*_*m*_. This approximation becomes better with increasing *N*_*m*_ (see Fig 5).

The transition region, which follows the fractal one, is specific to structures generated from CGR, and its length is inversely proportional with the number of scattering units. Since, from one hand the upper border of transition region (here, at about *qa* ≃ 9 * 10^2^) is fixed by the smallest distances inside the fractal, while from another hand, the lower border is fixed by the overall size of the fractal, then the decrease of the length of transition region is accomplished by the increase of length of fractal region (see Figs 3 and 5). Experimentally, from SAS data, such a behavior is a blueprint which can be used to identify structures generated from CGR.

The asymptotic region follows the transition one, and it’s characterized by a succession of maxima and minima which are damped out with increasing *q*. Their limiting values are shown by horizontal lines (see Figs 3 and 5), and they can be used to determine the number of units inside the fractal.

## 4 Applications

We apply the above findings to study the structural properties of single and multiscale fractals, and for which an analytical expression of the structure factor is not known. In the former case we consider a pentaflake fractal and a Cantor set with the same scaling factor *β*_s_ = 0.4, while in the latter one we consider the CGR of a DNA sequence.

### 4.1 Single scale fractals: Pentaflake and Cantor set

**Fig 6:**
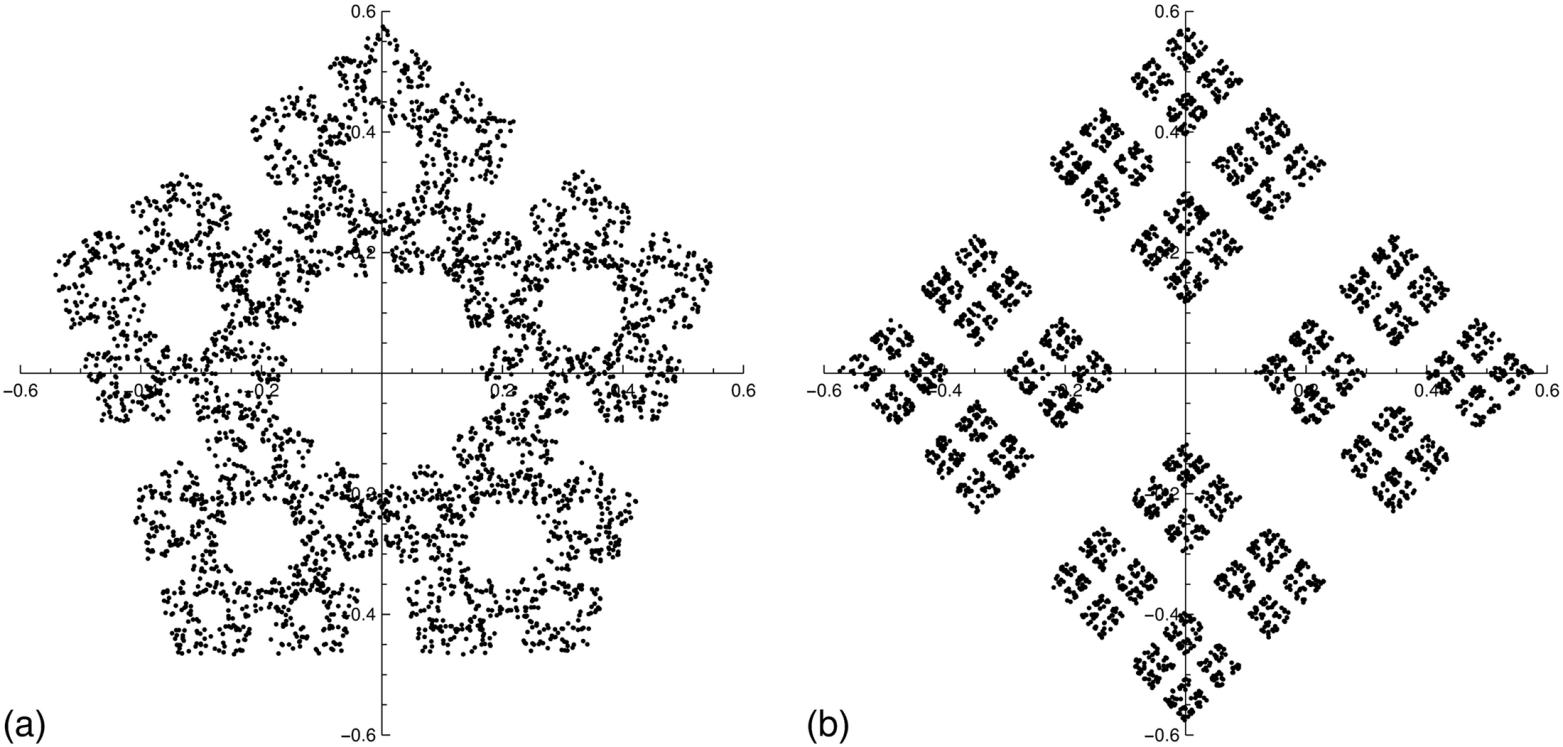
Single scale fractals obtained by CGR with *k* = 4000 points. (a) fractal pentaflake; (b) Cantor set.

The fractal pentaflake studied here is generated by starting with the center of an 5-sided polygon and the Cantor set is generated by starting with the center of a square. In both cases, a new point is drawn at a fraction *β*_s_ = 0.4 of the distance between the center and a randomly chosen vertex. Repeating the process for *k* = 4000 points, gives the structures shown in Fig 6a, and respectively in Fig 6b. Since, the fractal dimension of a CGR fractal generated using *n* affine transforms is

$$\begin{array}{c} D\approx -\log n/\log\beta_{s}, \end{array}$$ (21)

then the fractal dimensions of the pentaflake and Cantor set, are 1.76, and respectively 1.51.

**Fig 7:**
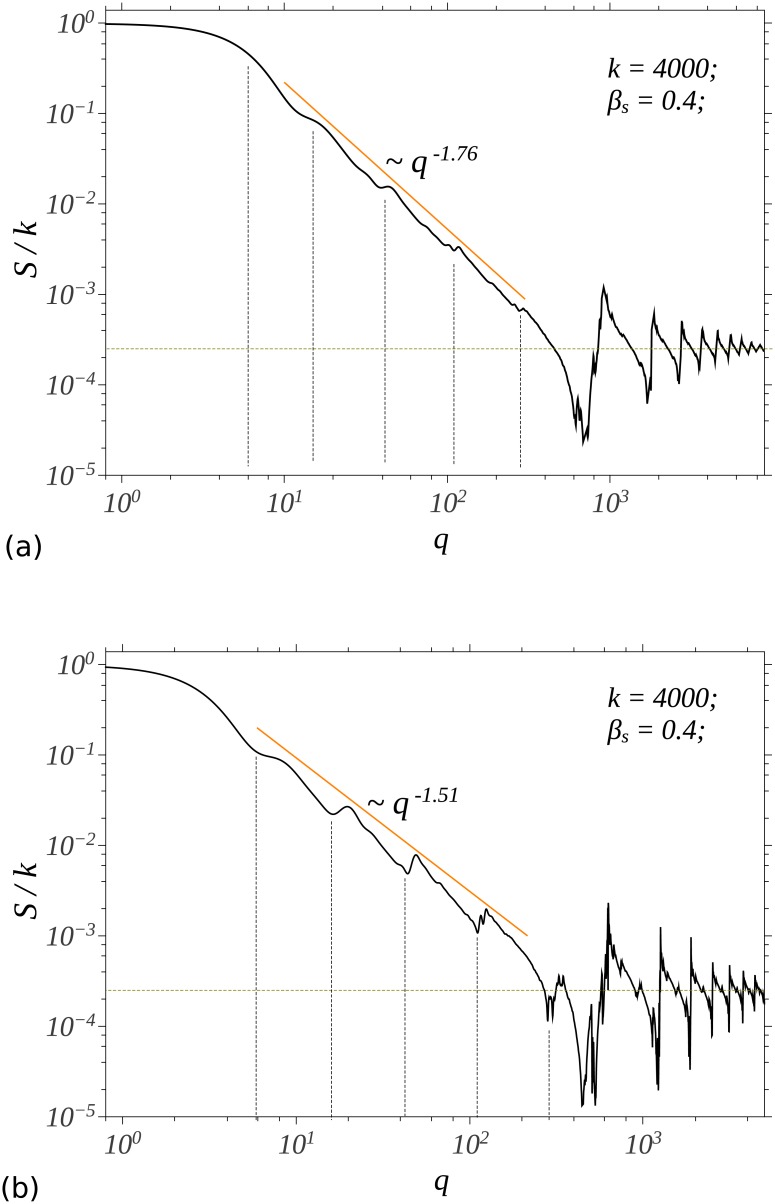
The structure factor of single scale fractals. (a) fractal pentaflake; (b) Cantor set.

The corresponding structure factors of the pentaflake and Cantor set calculated using Eq (20) are shown in Fig 7a and 7b. As expected, all the main features of SAS from CGR structures are preserved (see previous section) and the numerical value of the fractal dimension coincides with the one given by Eq (21). The value *β*_s_ = 0.4 of the scaling factor can be recovered from the periodicity of minima in the fractal region (see also Ref. [25]), and this is a specific feature of scattering from fractals with a single scale.

### 4.2 Multiscale fractals: DNA sequences

It is known that in some cases the chaos game can be used to display visually certain kinds of non-randomness. By considering the four bases “*A*”, “*C*”, “*G*” and “*T*” (or “*U*”) of DNA sequences, then the actual DNA can be used to control the chaos game by assigning the CGR vertices, to the four nucleotides labeled *A* = (−0.5, −0.5), *C* = (−0.5, 0.5), *G* = (0.5, 0.5), and *T* = (0.5, −0.5). Then, the CGR coordinates are obtained by plotting the first nucleotide in the sequence at half way between the center of the square *ACGT* and the corner with identical label. Next nucleotide is plotted half way between the point just plotted and the corner with identical label. Repeating the process for at least few thousand of times, produces a graphic with fractal patterns in the gene structures.

**Fig 8:**
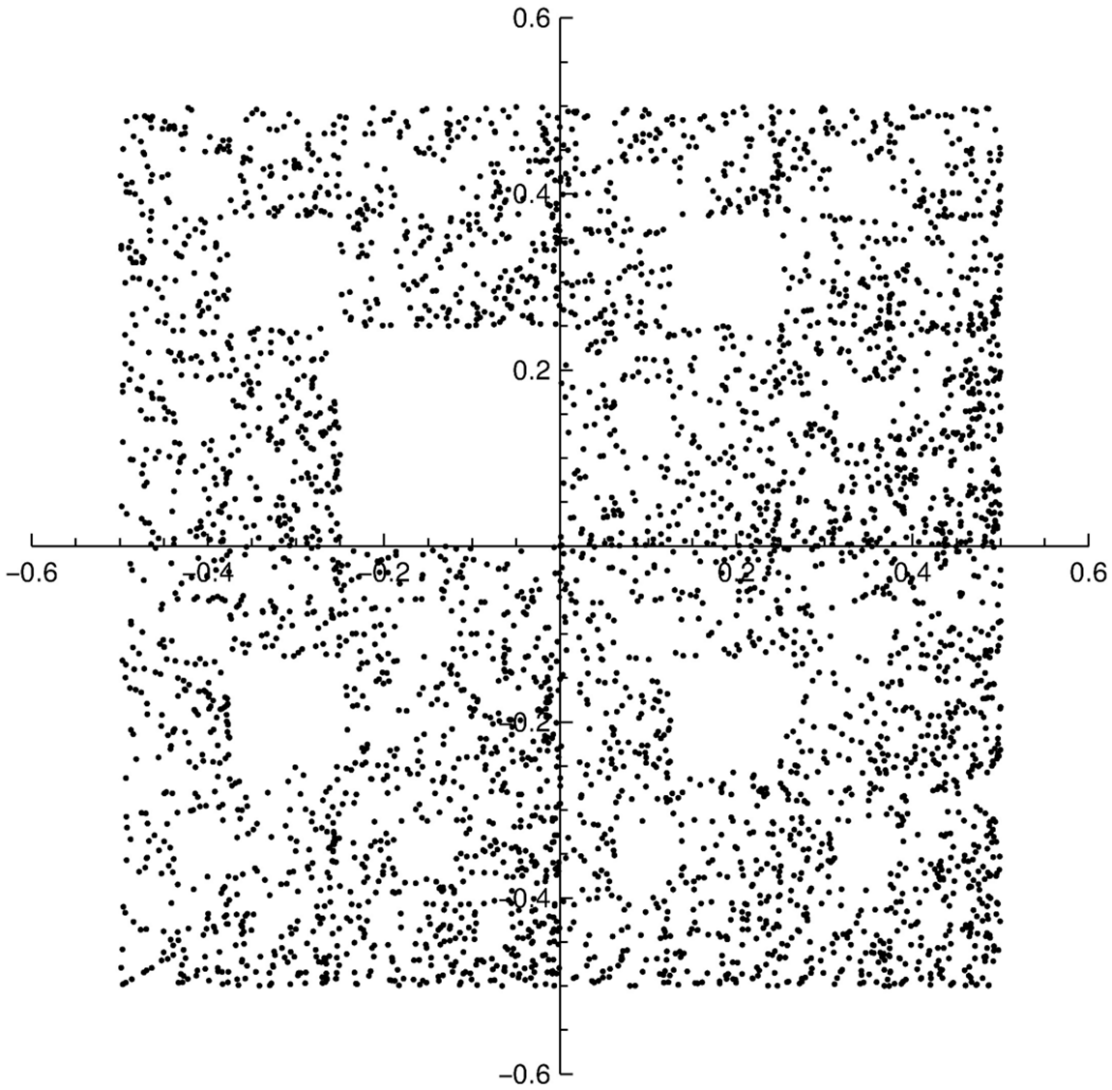
A random sequence of 4000 moves in the chaos game in the *ACGT* square when the sequence *GC* is eliminated.

Missing subsequences are found in a number of genetic sequences, such as human serum albumin or human adenosine deaminase genes, and a quick visualization of such patterns can be performed if some restrictions on the moves of chaos game are imposed [31]. Fig 8 shows the CGR of 4000 bases of a random sequence of moves in the square *ACGT*, where there is never a move toward vertex *G*, followed by a move toward vertex *C*, thus the sequence *GC* being eliminated.

**Fig 9:**
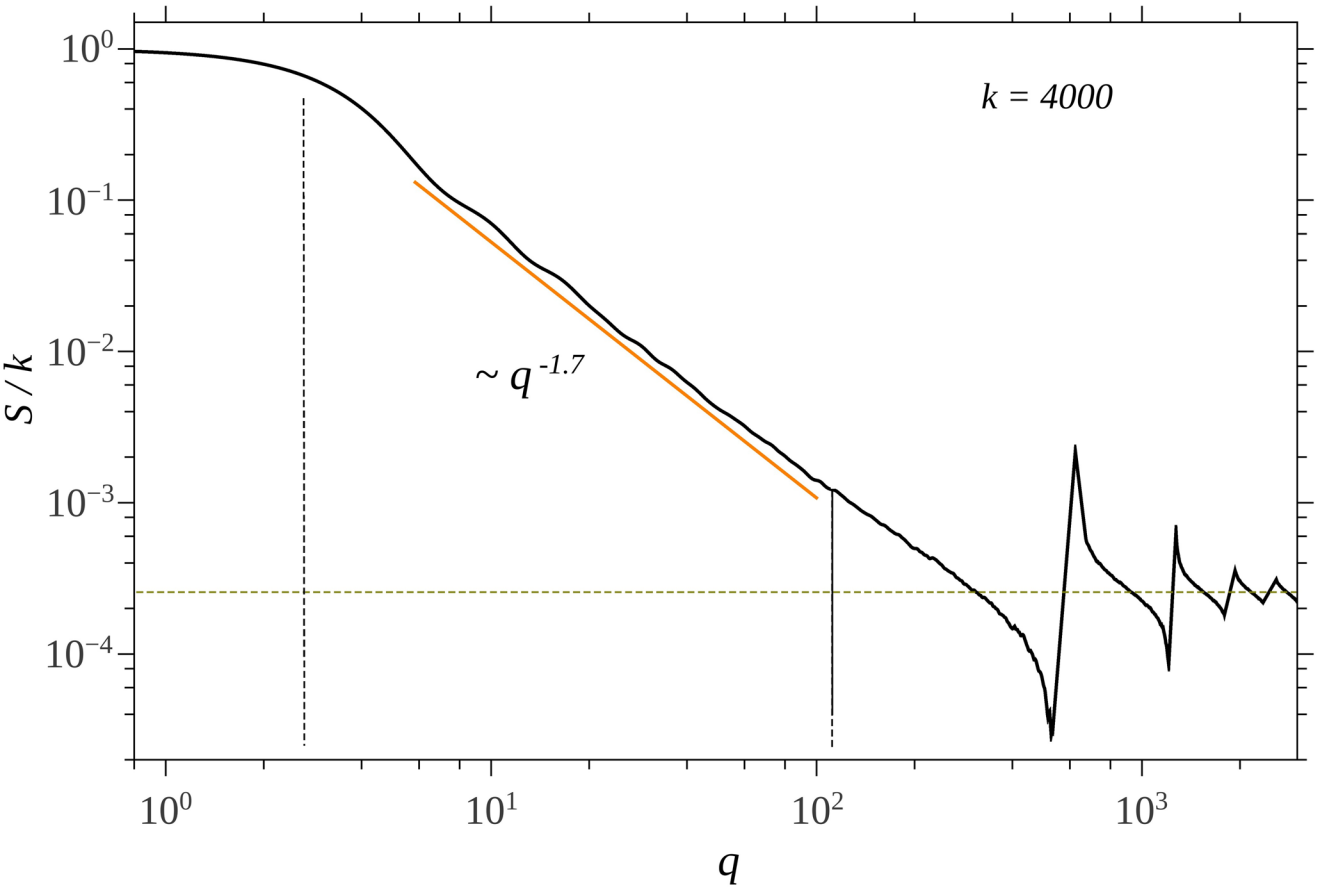
The corresponding structure factor of the CGR of DNA sequence shown in Fig 8.

By considering the coordinates of these bases as input coordinates in Eq (20), the corresponding SAS spectrum will have the characteristics shown in Fig 9. Although the scattering curve is characterized by the presence of a simple power-law decay, from which a fractal dimension can be obtained, there is no clearly visible a superposition of maxima and minima on a simple power-law decay, as in the case of the single scale fractals shown in Fig 6. We can attribute this feature to the presence of multiple scaling factors, which is a signature of the multifractal structure of the sequence.

## Conclusion

Structural properties of fractal structures generated using CGR are obtained from SAS intensity. An analytical expression for the scattering amplitude of 2D Sierpinski gasket is derived (see Eq 16).

It is shown that in the fractal region, the scattering curve of 2D Sierpinski gasket obtained from CGR reproduces very well the corresponding analytical curve, when the number of particles used in CGR is at least 4 or 5 times higher than the total number of particles in the deterministic case (see Fig 5). This is due to the random iteration algorithm used in generating spatial positions of scattering centers. Thus, in building a model based on CGR, this could serve as an indication of the minimum number of particles needed to approximate a given iteration in a deterministic fractal.

We show that for single scale mass fractals, the main properties such as the overall size, scaling factor, minimal distance between scattering units, fractal dimension, and the number of units can be recovered from SAS data. However, for multiscale fractals (fractals with multiple scaling factors) generated in the framework of CGR approach, the scattering curve does not show any clear periodicity in the fractal region, and thus none of the scaling factors can be recovered.

The main applications of the above findings can be found in investigating the structural properties of various deterministic nano and micro fractals obtained in last years, such as 2D Sierpinski hexagonal gasket [3], 2D Cantor sets [5], or 3D Koch-like fractals [7].
